# Increased circulating levels of SP-D and IL-10 are associated with the development of disease severity and pulmonary fibrosis in patients with COVID-19

**DOI:** 10.3389/fimmu.2025.1553283

**Published:** 2025-03-14

**Authors:** Xin Pan, Zhisheng Huang, Ningning Tao, Chuanjun Huang, Shanshan Wang, Zuowang Cheng, Ruyue Fan, Shuai Liu

**Affiliations:** ^1^ Department of Respiratory and Critical Care Medicine, Shandong Provincial Hospital Affiliated to Shandong First Medical University, Jinan, Shandong, China; ^2^ Department of Pulmonary and Critical Care Medicine, The First Affiliated Hospital of Nanchang University, Nanchang, Jiangxi, China; ^3^ Department of Pulmonary and Critical Care Medicine, National Regional Center for Respiratory Medicine, Jiangxi hospital of China-Japan Friendship Hospital, Nanchang, Jiangxi, China; ^4^ Department of Laboratory Medicine, Shandong Provincial Hospital Affiliated to Shandong First Medical University, Jinan, Shandong, China; ^5^ Department of Clinical Laboratory, Zhangqiu District People’s Hospital Affiliated to Jining Medical University, Jinan, Shandong, China; ^6^ Shandong Center for Disease Control and Prevention, Jinan, Shandong, China; ^7^ Shandong Xinhua Pharmaceutical Co., Ltd, Zibo, Shandong, China

**Keywords:** COVID-19, acute respiratory distress syndrome, pulmonary fibrosis, surfactant protein-d, interleukin-10, angiotensin-converting enzyme-2

## Abstract

**Background:**

Patients with severe COVID-19 can rapidly develop acute respiratory distress syndrome (ARDS), which further increases the risk of developing pulmonary fibrosis. The exact role of macrophage polarization and different cytokine production in the pathophysiology associated with COVID-19 induced ARDS or pulmonary fibrosis is unknown. It is necessary to identify potential biomarkers that can predict the progress of pulmonary fibrosis or other adverse consequences.

**Methods:**

We analyze the plasma samples obtained from healthy individuals and COVID-19 patients who were stratified according to the disease severity and fibrotic-like changes on chest computed tomography (CT) scans. Surfactant Protein D (SP-D), Matrix Metalloproteinase 8 (MMP8), Krebs von den lungen-6 (KL-6), Angiotensin‐Converting Enzyme 2 (ACE2), and macrophage polarization-related biomarkers were determined by ELISA. Data were collected and evaluated using regression models and receiver operating characteristic (ROC) curves.

**Results:**

The plasma levels of SP-D, MMP8 in patients with ARDS were higher than those of non-ARDS patients. Patients with pulmonary fibrosis had higher plasma levels of SP-D compared to those without fibrotic changes. Among the biomarkers indicative of macrophage polarization, compared to non-ARDS patients, a significant increase in IL-10, Inducible nitric oxide synthase (iNOS), and Arginase-1 (Arg-1) were observed in ARDS patients, while Tumor necrosis factor-α (TNF-α) was decreased. The plasma level of IL-10 was also elevated in patients with fibrotic changes on CT, and was positively correlated with ACE2 and Arg-1. ROC curve results uncovered that SP-D showed higher efficacy in predicting pulmonary fibrosis and ARDS compared to other inflammatory markers. And IL-10 had similar predictive value with traditional inflammatory indicators such as CRP and PCT.

**Conclusion:**

SP-D and IL-10 exhibited certain predictive abilities for the development of ARDS and pulmonary fibrosis in patients with COVID-19. The determination of these cytokines upon admission is crucial for evaluating the prognosis of COVID-19 patients.

## 1 Introduction

Coronavirus disease 2019 (COVID-19) caused by severe acute respiratory syndrome coronavirus 2 (SARS-CoV-2) remains a pandemic, with infections or reinfections occurring worldwide (1). The infection imposes a substantial burden on patients with preexisting pulmonary diseases, promoting the decline in lung function and exacerbating respiratory symptoms (2, 3). Patients with severe COVID-19 could rapidly progress to acute respiratory distress syndrome (ARDS) with hypoxemic respiratory failure, requiring lung-protective ventilation strategies (4). These further heighten the risk of developing lung fibrosis and accelerate the progression of existing fibrotic lung diseases, particularly in elderly populations (5).

The interaction between SARS-CoV-2 and the host’s immune response frequently leads to persistent inflammation, while the excessive expression of pro-inflammatory cytokines further contributes to endothelial dysfunction and tissue damage, thereby creating an environment conducive to pulmonary fibrosis (6). The exact prevalence of post-COVID-19 related pulmonary fibrosis is unknown, but it is associated with older age, disease severity, mechanical ventilation, and ARDS (7, 8). The occurrence of ARDS associated pulmonary fibrosis is strongly associated with poor prognosis, which includes prolonged mechanical ventilation, increased mortality, and persistent lung abnormalities following discharge (9, 10). However, it remains unclear why the majority of COVID-19 patients with ARDS are able to recover from the initial inflammatory storm, while a subset of patients exhibit evident and persistent radiological lung abnormalities, including fibrotic lung changes that occur early in the disease. Given the heterogeneous prognosis of COVID-19 patients, identifying potential biomarkers that can predict the progression of pulmonary fibrosis or other adverse outcomes facilitates therapeutic intervention and monitoring of vulnerable populations within an optimal time window.

Pulmonary fibrosis, a late complication of ALI/ARDS, is characterized by fibroblast proliferation and excessive deposition of extracellular matrix. Evidence that traditional biomarkers may provide novel ideas for predicting disease progression and prognosis is progressively accumulating. Elevated krebs von den lungen-6 (KL-6) levels mainly reflect the occurrence of damage and subsequent regeneration in type II alveolar epithelial cells (11, 12). The surfactant protein-D (SP-D) has been identified as a promising biomarker to quantify epithelial damage and is elevated in patients with direct ARDS or interstitial lung diseases (ILDs) (13, 14). Previous studies have shown that MMP8 contributes to lung fibrotic responses after injury (15, 16). Human Angiotensin‐Converting Enzyme 2 (ACE2), as classical components of the RAS system, could also play a significant role in the pathogenesis of acute lung injury and ARDS. ACE2 activity presumably attenuates the immune response and protects against exaggerated inflammation associated with cytokine storm, profound tissue alterations, and the fibrotic remodeling of the lungs (17). However, the exact function of these biomarkers in the pathogenesis of COVID-19 associated with ARDS or pulmonary fibrosis remains ambiguous.

Viral infections have been identified as a significant major contributor to pulmonary fibrosis, with considerable effects on disease progression and outcomes (18, 19). Many research has elucidated the association between the above pulmonary-related proteins and prevalent respiratory infections (20). Current research emphasizes the role of alveolar macrophages in the process of pulmonary inflammatory injury and fibrosis (21). According to previous studies, SARS-CoV-2 causes lung tissues and trigger pulmonary fibrosis thought by an abnormal immune response, which results in over-activation of inflammatory cells such as macrophages, neutrophils, eosinophils, and T helper cells, the release of various pro-inflammatory and profibrotic cytokines, and the expansion of fibroblasts, specifically myofibroblasts (22, 23). This cascade ultimately leads to the persistence of lung damage and fibrosis. Furthermore, the multiple functions of macrophages are attributed to their activation heterogeneity, which is a stimulus-driven polarization process. Classic-activated M1 macrophages have cytotoxicity and promote the inflammatory process to participate in inflammatory pathological damage, while alternative-activated M2 can inhibit inflammation and repair wounds (24). The relationship between SARS-CoV-2 induced ARDS or pulmonary fibrosis and macrophage polarization still needs further investigation.

Here, we first discussed the expression of biological markers associated with pulmonary fibrosis and their potential significance in ARDS related fibrosis. Secondly, the relationship between the degree of fibrosis and the polarization of macrophages was further analyzed. Finally, the important role of key factors in the early recognition of ARDS and the development of pulmonary fibrosis was identified. Together, these studies will help elucidate new mechanisms underlying the development of fibrosis in severe COVID-19.

## 2 Materials and methods

### 2.1 Clinical samples

Whole-blood samples were collected from patients with COVID-19 from December 2022 to February 2023 admitted in department of pulmonary and critical care medicine of Shandong Provincial Hospital, Jinan, China. And only one blood sample was collected from each patient on an empty stomach within 24 h of admission. All subjects and patients were diagnosed with pneumonia or pulmonary fibrosis by chest CT scans, as described below. The throat swab samples were tested by real-time polymerase chain reaction (RT-PCR) to detect the presence of SARS-CoV-2. The arterial blood gas, arterial partial pressure of oxygen (PaO2) and the fraction of inspired oxygen (FiO2) from COVID-19 patients were collected. The diagnosis of ARDS used was based on the Berlin definition of ARDS (25). Twenty-five peripheral blood samples from healthy subjects was collected according to negative swab to SARS-CoV-2. The plasma was isolated from whole blood samples and flash-frozen and stored at -80°C respectively until using.

### 2.2 Chest CT Scans and image evaluation

All the patients were imaged with a multi-detector HiSpeed Dual CT scanner. The scans encompassed the region from the upper thoracic inlet to the inferior costophrenic angle. All CT images were independently and randomly reviewed by three radiologists who had five years of experience, and the resolutions of any disagreements were achieved through discussion and consensus. The predominant chest CT images were enumerated as follows: ground glass opacity (GGO), consolidation, reticulation, septal thickening and/or reticulation, linear bands, thickening of the bronchial wall, nodules, bronchiectasis and interlobar pleural traction, among others. The chest CT evidence of fibrotic-like changes was defined as a combination of findings, including irregular septal thickening, reticulation or honeycombing, and traction bronchiectasis (26, 27). Patients were divided into two groups according to the evidence of fibrotic-like lung changes on their CT images.

### 2.3 Enzyme-linked immunosorbent assay

The concentration levels of Human Surfactant Protein D (SP-D), Human Matrix Metalloproteinase 8 (MMP8), Human Krebs von den Lungen-6 (KL-6), Human Angiotensin‐Converting Enzyme 2 (ACE2), Human Interleukin-10 (IL-10), Human Arginase-1 (Arg-1), Human Transforming Growth Factor-β (TGF-β), Human Vascular Endothelial-derived Growth Factor (VEGF), Human Inducible nitric oxide synthase (iNOS) and Human Tumor necrosis factor-α (TNF-α), Human Interleukin-1β (IL-1β), Human Interleukin-12 (IL-12) in the plasma samples were measured according to the manufacturer’s instructions (Elabscience Company, USA). We analyzed the difference of biomarkers expression between healthy donors and COVID-19 patients with different severity or degree of pulmonary fibrosis.

### 2.4 Statistical analysis

Statistical analysis was performed with Graph-Pad Prism software (version 5.0) and statistical significance was calculated by Student’s *t*-test, Mann-Whitney *U* test, χ2 test, or Fisher’s exact test, as appropriate. The predictive accuracy of the biomarkers was calculated using ROC curve analysis. *P < 0.05, **P < 0.01, and ***P < 0.001 represent significant differences.

## 3 Results

### 3.1 Clinical and demographical features of patients with COVID-19

The study included 112 hospitalized patients with COVID-19 and 25 healthy controls. As shown in **Table 1**, the non-ARDS group and ARDS group consisted of 46 and 66 patients with COVID-19, respectively. All patients with COVID-19 enrolled had at least one comorbidity. The total leukocyte count, neutrophil count, and proportion of neutrophils were significantly higher in ARDS patients compared with patients with non-ARDS, while the proportion of lymphocytes was lower. The highest-level respiratory support strategy was summarized. In all patients with COVID-19, 93.75% of patients (105 of 112) required supplemental oxygen. In the ARDS group, 46.97% (31 of 66) need superior respiratory support (such as high-flow nasal cannula oxygen therapy or mechanical ventilatory support). The clinical information for patients with COVID-19, classified by whether pulmonary fibrosis has occurred, is shown in **Table 2**. Among the 112 patients included in this study, fibrotic-like changes in varying degrees (based on the fibrous lesions shown on the patients’ first radiological examination upon admission) were observed on 44.64% (50 of 112) of chest CT scans during hospitalization. No significant baseline demographic differences were observed between the PF and no-PF groups other than age in both cohorts.

**Table 1:** Characteristics of all patients admitted to the hospital for COVID-19.

	Total (*n* = 112)	Non-ARDS (*n* = 46)	ARDS (*n* = 66)	*p* value
Gender				0.0018^a^
Male	68 (60.71%)	20 (43.48%)	48 (72.73%)	
Female	44 (39.29%)	26 (56.52%)	18 (27.27%)	
Age/years (mean (SD))	70.60 (14.16)	66.30 (15.31)	73.59 (12.58)	0.0068^b^
Comorbidity				
Pneumonia disease	112 (100%)	46 (100%)	66 (100%)	-
Cancer	20 (17.86%)	6 (13.04%)	14 (21.21%)	0.2668^a^
Hypertension	49 (43.75%)	14 (30.43%)	35 (53.03%)	0.0177^a^
Diabetes	24 (21.43%)	9 (19.57%)	15 (22.73%)	0.6883^a^
Coronary heart disease	38 (33.93%)	15 (32.61%)	23 (34.85%)	0.8055^a^
Cerebrovascular disease	20 (17.86%)	8 (17.39%)	12 (18.18%)	0.9144^a^
Laboratory tests (mean (SD))				
Total leukocytes (×10^9^/L)	7.98 (3.87)	6.94 (3.74)	8.72 (3.81)	0.0161^b^
Neutrophil (×10^9^/L)	6.45 (3.73)	5.26 (3.55)	7.28 (3.64)	0.0042^b^
Proportion of Neutrophil (%)	77.43 (13.97)	71.44 (14.30)	81.60 (12.18)	0.0000^b^
Lymphocytes (×10^9^/L)	1.01 (0.68)	1.15 (0.75)	0.91 (0.60)	0.0551^b^
Proportion of Lymphocytes (%)	16.79 (14.35)	21.59 (14.37)	13.44 (13.45)	0.0027^b^
Platelet (×10^9^/L)	207.66 (95.47)	215.35 (84.44)	202.30 (102.75)	0.4793^b^
C-reactive protein (mg/l)	35.82 (47.49)	28.62 (43.59)	41.16 (49.85)	0.1758^b^
Procalcitonin (ng/ml)	0.15 (0.23)	0.11 (0.10)	0.18 (0.28)	0.1232^b^
Interleukin-6 (pg/ml)	29.03 (74.84)	14.33 (23.94)	38.53 (93.15)	0.1085^b^
Radiologic characteristic				
Fibrotic-like changes on Chest CT (%)	50 (44.64%)	9 (19.57%)	41 (62.12%)	0.0000^a^
PaO_2_/FiO_2_ grade (mmHg)				
>300	46 (41.07%)	46 (100%)	0	0.0000^a^
>200 to ≤300	33 (29.46%)	0	33 (50.00%)	0.0000^a^
>100 to ≤200	26 (23.22%)	0	26 (39.39%)	0.0000^a^
≤100	7 (6.25%)	0	7 (10.61%)	0.0400^c^
Respiratory support				
Invasive mechanical ventilation	2 (1.79%)	0	2 (3.03%)	0.5116^c^
Noninvasive mechanical ventilatory support	5 (4.46%)	0	5 (7.58%)	0.0768^c^
High-flow nasal cannula oxygen therapy	25 (22.32%)	1 (2.17%)	24 (36.36%)	0.0000^a^
Low-flow oxygen therapy	73 (65.18%)	38 (82.06%)	35 (53.03%)	0.0012^a^

All the participants resided in Shandong, China. Data are mean (SD) or n (%). *p* values were calculated by Student’s t test, chi-squared test, or Fisher’s exact test, as appropriate.

a Compared by two-sided chi-squared test.

b Compared by two-sided Student’s *t* test.

c Compared by two-sided Fisher’s exact test.

**Table 2:** Clinical characteristics of the patients with COVID-19 classified by the pulmonary fibrosis.

	No fibrotic-like changes on CT (*n* = 62)	Fibrotic-like changes on CT (*n* = 50)	*p* value
Gender			0.0002^a^
Male	28 (45.16%)	40 (80.00%)	
Female	34 (54.84%)	10 (20.00%)	
Age/years (mean (SD))	66.95 (16.58)	75.12 (8.63)	0.0021^b^
Comorbidity			
Cancer	10 (16.13%)	10 (20.00%)	0.5949^a^
Hypertension	25 (40.32%)	24 (48.00%)	0.4155^a^
Diabetes	15 (24.19%)	9 (18.00%)	0.4271^a^
Coronary heart disease	18 (29.03%)	20 (40.00%)	0.2230^a^
Cerebrovascular disease	9 (14.52%)	11 (20.00%)	0.3039^a^
Laboratory tests (mean (SD))			
Total leukocytes (×10^9^/L)	7.73 (3.96)	8.30 (3.76)	0.4445^b^
Neutrophil (×10^9^/L)	6.13 (3.77)	6.86 (3.67)	0.3049^b^
Proportion of Neutrophil (%)	75.63 (13.79)	79.65 (14.00)	0.1305^b^
Lymphocytes (×10^9^/L)	1.04 (0.70)	0.98 (0.65)	0.6044^b^
Proportion of Lymphocytes (%)	17.67 (13.66)	15.70 (15.22)	0.4734^b^
Platelet (×10^9^/L)	202.87 (88.47)	213.60 (104.11)	0.5567^b^
C-reactive protein (mg/l)	35.80 (48.87)	35.83 (46.15)	0.9975^b^
Procalcitonin (ng/ml)	0.14 (0.15)	0.17 (0.30)	0.5158^b^
Interleukin-6 (pg/ml)	28.56 (89.45)	29.58 (54.52)	0.9186^b^

Data are mean (SD) or n (%). *p* values were calculated by Student’s t test, chi-squared test, as appropriate.

a Compared by two-sided chi-squared test.

b Compared by two-sided Student’s *t* test.

### 3.2 The expression of SP-D and MMP8 in plasma was elevated in ARDS patients with COVID-19

**Figure 1:**
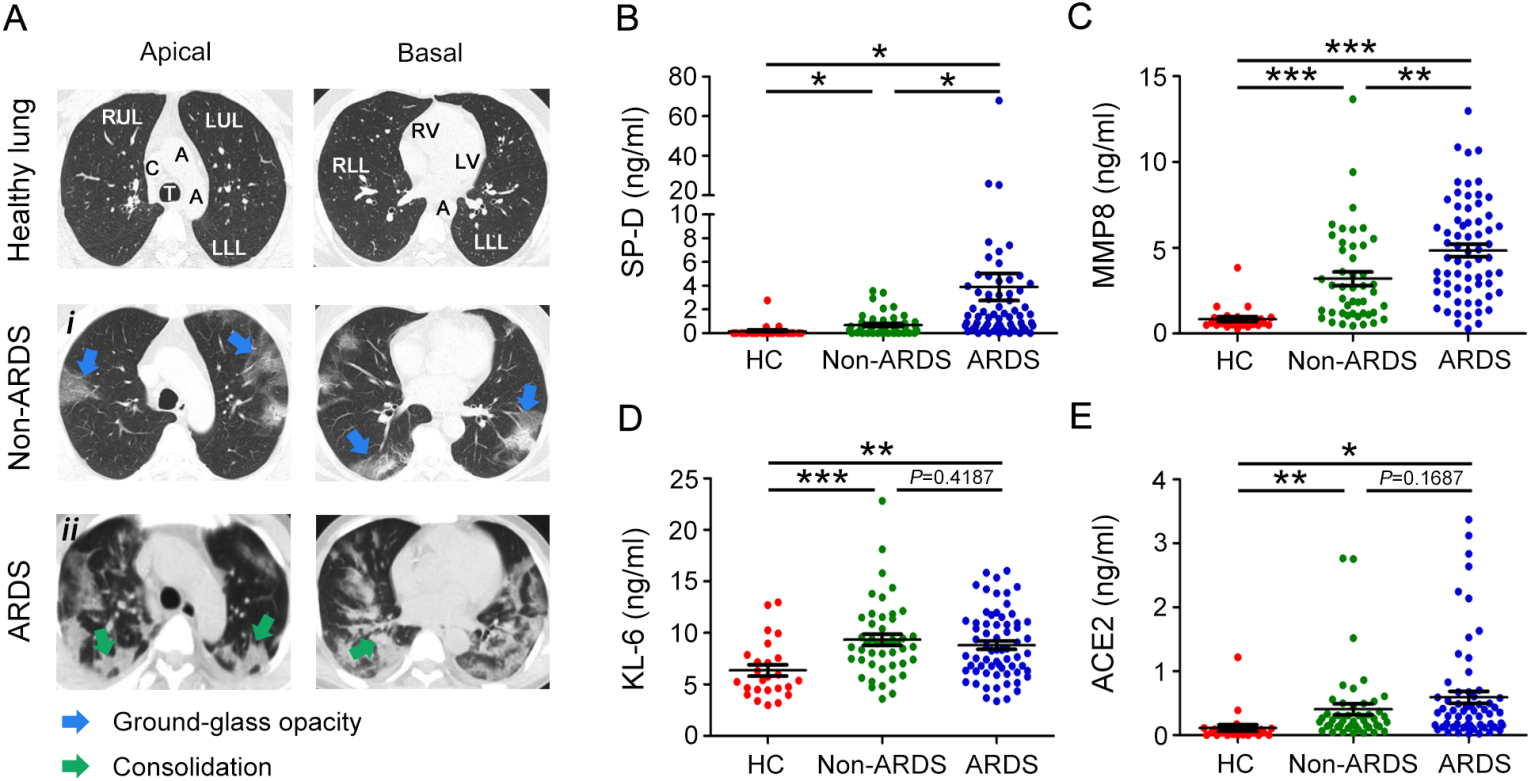
Evaluation of chest CT image and plasma SP-D, MMP8, KL-6, and ACE2 levels in COVID-19 patients and healthy donors. **(A)** Multiple axial sections of CT chest lung windows of ARDS and non-ARDS patients with COVID-19 showing the imaging spectrum of bilateral pneumonia. CT scan shows ground glass opacities (blue arrowheads) or consolidation (green arrowheads). Evaluation of plasma levels of **(B)** SP-D, **(C)** MMP8, **(D)** KL-6, and **(E)** ACE2 in patients with ARDS (ARDS group), patients without ARDS (non-ARDS group), and health controls (HC). The data are displayed as mean ± SD. Statistical significance is determined by Unpaired *t* test, and Mann-Whitney U test. *P < 0.05, **P < 0.01, ***P < 0.001. SP-D, surfactant protein D; MMP8, matrix metalloproteinase 8; KL-6, Krebs von den Lungen-6; ACE2, angiotensin‐converting enzyme 2.

The chest CT images were presented in **Figure 1A**. The axial CT scan obtained from non-ARDS patients revealed multiple strip-like ground-glass opacities in bilateral lungs (**Figure 1A**i); Whereas the CT scan obtained from ARDS patients exhibited bilateral diffuse consolidation in bilateral lungs and flake-like ground-glass opacity can be observed in areas without consolidation, which is also called the “white lung” sign (**Figure 1A**ii). SP-D, MMP8, and KL-6 are associated with acute lung disease. To assess the diagnostic value of these biomarkers and understand whether they could predict the severity of pulmonary disease in COVID-19 patients, plasma levels of SP-D, MMP8 and KL-6 were measured in the ARDS and non-ARDS groups. We found that plasma SP-D was statistically higher in COVID-19 patients than in healthy subjects. The level of SP-D was notably higher in the ARDS group when compared with the non-ARDS group (**Figure 1B**). Furthermore, the level of MMP8 was significantly higher in ARDS patients when compared to non-ARDS patients (**Figure 1C**). In sharp contrast, we found that plasma levels of KL-6 in ARDS patients were not statistically different than in non-ARDS groups (**Figure 1D**). Previous clinical studies showed that ACE2 could impact the severity of ARDS. In this regard, we found that COVID-19 patients had higher levels of ACE2 compared to healthy subjects. However, no statistical differences were observed between non-ARDS and ARDS patients (**Figure 1E**). These results underlined the potential role of plasma SP-D and MMP8 evaluation to detect and monitor acute lung damage associated with COVID-19.

### 3.3 Plasma levels of SP-D were elevated in patients with COVID-19 who had pulmonary fibrotic-like events

**Figure 2:**
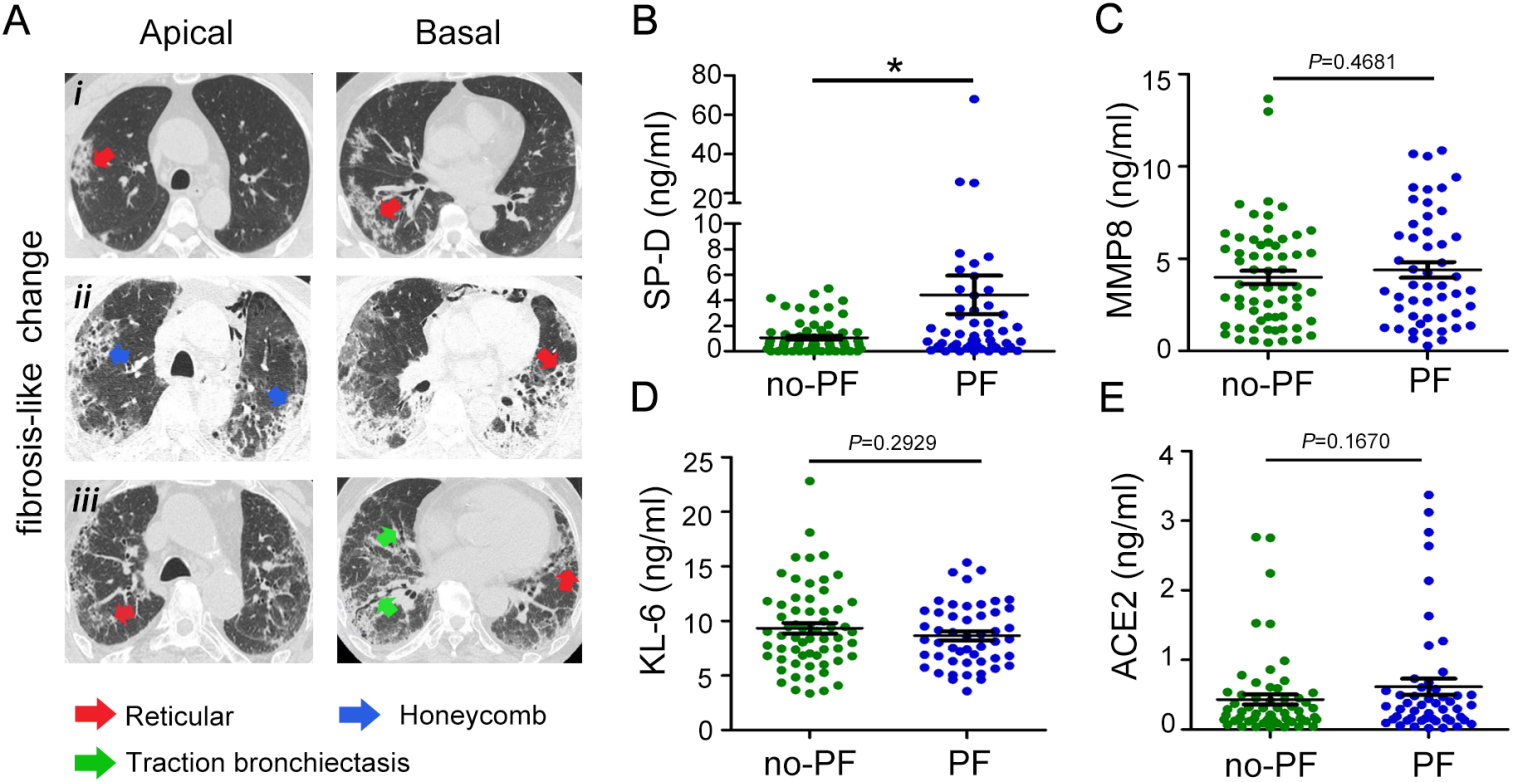
Evaluation of SP-D, MMP8, KL-6, and ACE2 levels in patients with COVID-19 with fibrosis-like chest CT images. **(A)** Chest CT scans of patients with COVID-19 showed reticular (red arrowheads), honeycomb-like pattern (blue arrowheads), and traction bronchiectasis changes (green arrowheads). **(B)** Evaluation of plasma levels of **(B)** SP-D, **(C)** MMP8, **(D)** KL-6, and **(E)** ACE2 in patients with COVID-19 stratified according to pulmonary fibrosis. The data are displayed as mean ± SD. Statistical significance is determined by Unpaired *t* test. *P < 0.05.

We stratified COVID-19 patients as fibrotic-like (PF) or no fibrotic-like (no-PF) changes groups according to whether they had signs of fibrosis on chest CT upon admission. As shown in **Figure 2A**. Most of the lungs of patients with signs of fibrosis exhibited reticular and thickening of the adjacent pleura, localized honeycombing in the subpleural region, and mild cylindrical traction bronchiectasis (**Figure 2A**i-iii). SP-D, MMP8 and KL-6 are also well-studied as specific pulmonary fibrosis-associated biomarkers. Patients with fibrotic-like changes groups had significantly higher levels of SP-D than healthy subjects and patients with no fibrotic-like changes (**Figure 2B**). However, the stratification of COVID-19 patients as no-PF versus PF did not show any difference in MMP8, and KL-6 levels (**Figures 2C, D**). SARS-CoV-2 can bind with ACE2 and activate fibrosis-related genes and processes to induce lung fibrosis. As for plasma levels of ACE2, no statistically significant differences were observed between the non-PF and PF groups (**Figure 2E**). Overall, these data demonstrated that SP-D was most closely associated with the development of pulmonary fibrosis in patients with COVID-19.

### 3.4 Various macrophage polarization-related cytokines could be indicative of ARDS or pulmonary fibrosis caused by COVID-19

**Figure 3:**
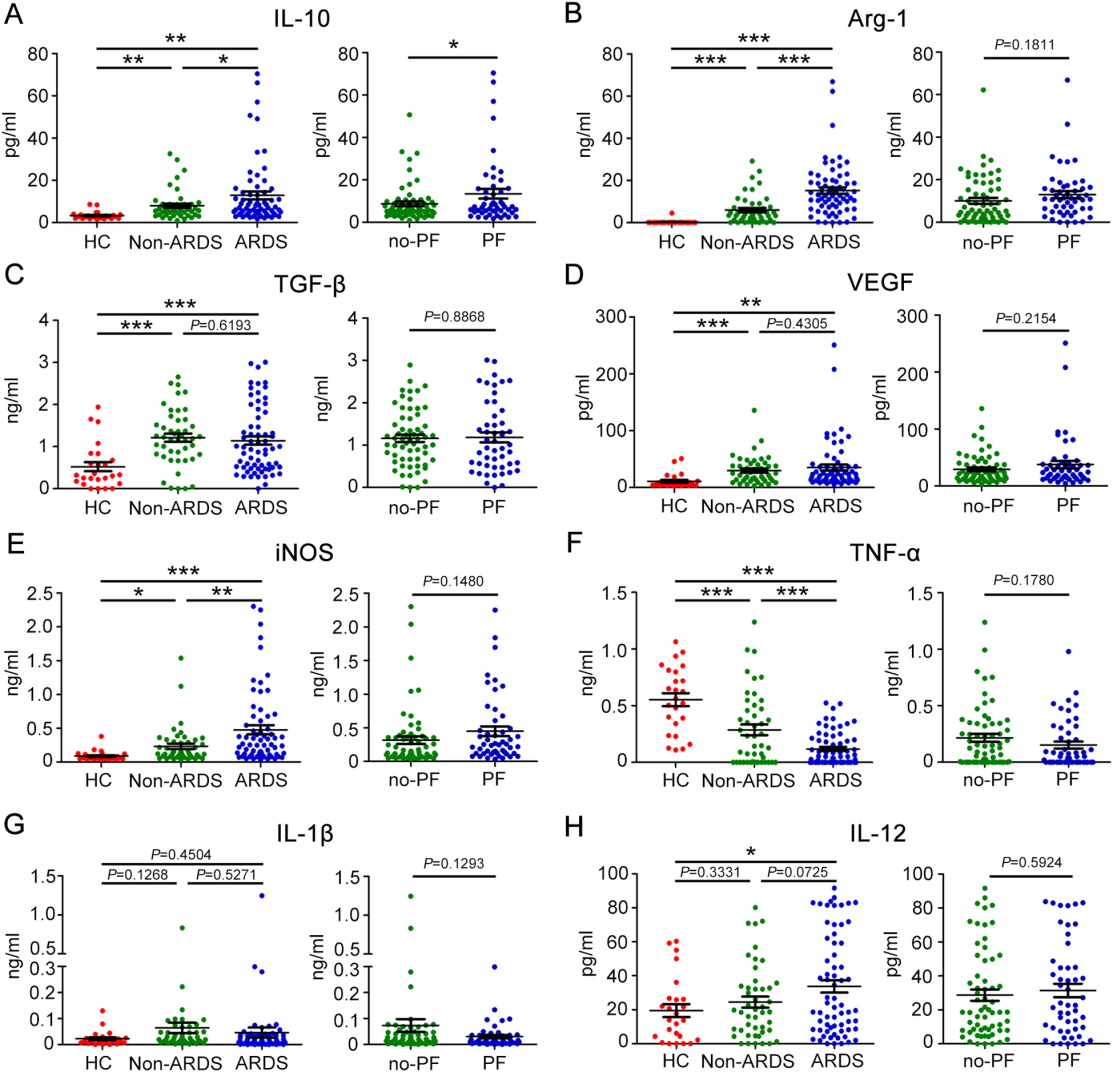
Plasma levels of eight biomarkers associated with M1 or M2-like macrophage polarization in patients with COVID-19. Baseline plasma levels of biomarkers associated with M2-like macrophage polarization in COVID-19 patients with different severity and degrees of lung fibrosis. **(A)** IL-10, **(B)** Arg-1, **(C)** TGF-β, and **(D)** VEGF. Baseline plasma levels of biomarkers associated with M1-like macrophage polarization in patients with COVID-19. **(E)** iNOS, **(F)** TNF-α, **(G)** IL-1β, and **(H)** IL-12. Statistical significance is determined by Unpaired *t* test, and Mann-Whitney U test. *P < 0.05, **P < 0.01, ***P < 0.001. IL-10, Interleukin-10; Arg-1, Arginase-1; TGF-β, Transforming growth factor-β; VEGF, vascular endothelial-derived growth factor; iNOS, inducible nitric oxide synthase; TNF-α, Tumour Necrosis Factor-α; IL-1β, Interleukin-1β; IL-12, Interleukin-12.

The balance between M1 and M2 macrophages is thought to play a role in regulating the trend and severity of lung fibrosis. We further characterized macrophage M2-type polarization in COVID-19 patients by measuring the expression levels of IL-10, Arg-1, TGF-β, and VEGF in plasma. We found that ARDS patients had higher levels of IL-10 compared to non-ARDS patients and healthy controls. Patients who had fibrotic-like changes on chest CT had higher levels of IL-10 than those who had no fibrotic-like changes (**Figure 3A**). The plasma level of Arg-1 increased with disease severity. Patients who had fibrotic-like changes showed a tendency to have higher levels of Arg-1 than those who had no fibrotic-like changes, although no statistical difference was observed between the two groups (**Figure 3B**). However, in terms of TGF-β and VEGF, no or weak differences were observed when plotted against patients with ARDS or fibrotic-like changes (**Figures 3C, D**). Furthermore, the expression levels of cytokines associated with M1 macrophages, such as iNOS, TNF-α, IL-1β, and IL-12, were subjected to measured. We observed that the plasma levels of iNOS were significantly increased in ARDS patients compared to non-ARDS patients and healthy subjects. In contrast, the plasma levels of TNF-α decreased with disease severity. Of note, with regard to iNOS and TNF-α, no statistical difference was observed between the fibrotic-like changes group and the no fibrotic-like changes group (**Figures 3E, F**). We found that plasma levels of IL-1β and IL-12 in COVID-19 patients were not statistically different than in healthy controls. Similarly, the stratification of COVID-19 patients as fibrosis versus no-fibrosis did not show any difference in IL-1β and IL-12 levels (**Figures 3G, H**). These data implied the importance of macrophages polarization in predicting COVID-19 severity and the development of pulmonary fibrosis.

### 3.5 Relationship among macrophages polarization-related cytokines and pulmonary damage or fibrosis biomarkers

**Figure 4:**
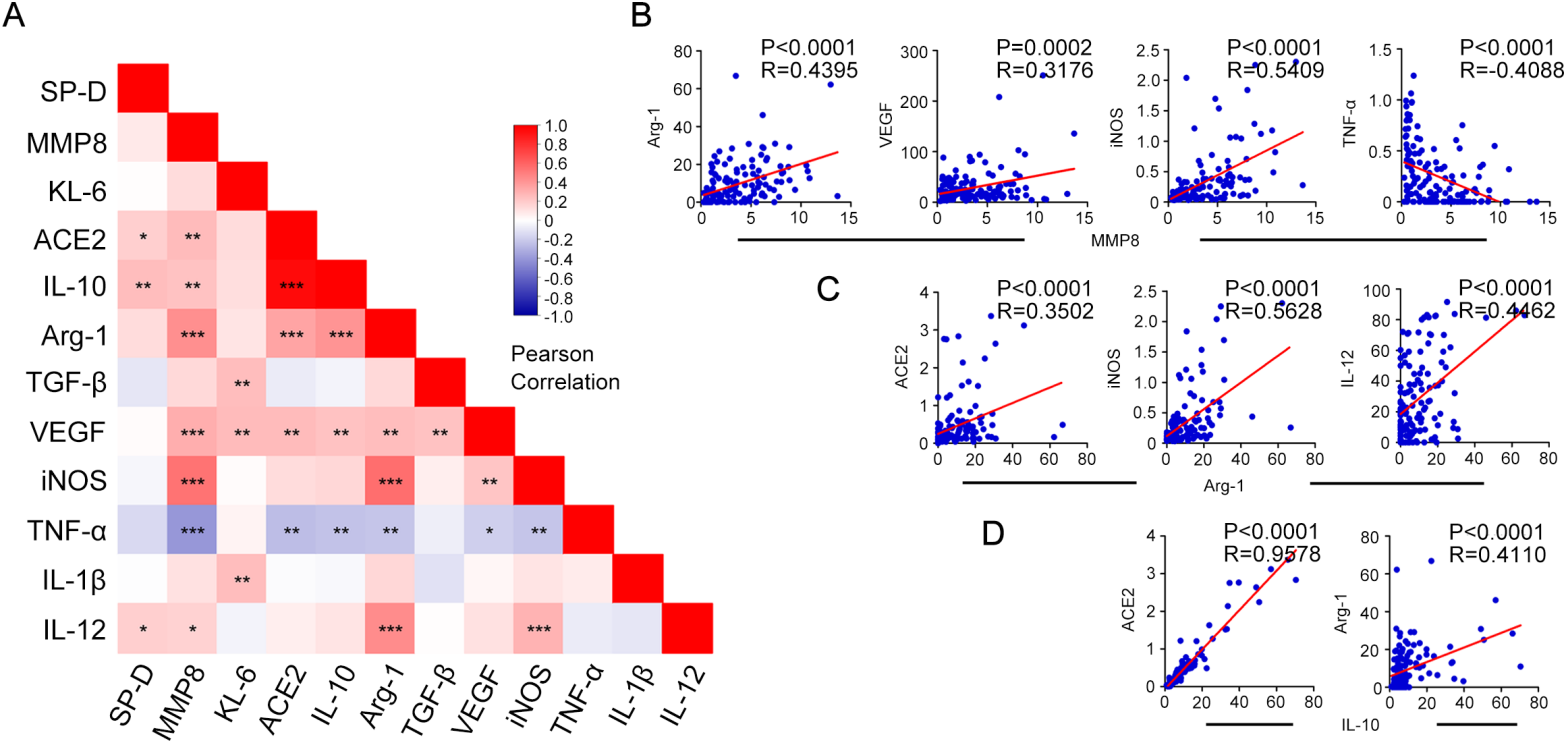
Spearman’s correlation analysis of key biomarkers associated with pulmonary fibrosis and macrophage polarization with each other. **(A)** Correlation-based heatmap demonstrating the association among the measured biomarkers associated with pulmonary fibrosis and macrophage polarization in patients with COVID-19. **(B)** Correlation analysis and scatter plot of the expression levels of MMP8 and key biomarkers Arg-1, VEGF, iNOS, and TNF-α in the plasma of patients with COVID-19. **(C)** Correlation analysis and scatter plot of the expression levels of Arg-1 and key biomarkers ACE2, iNOS, and IL-12 in the plasma of patients with COVID-19. **(D)** Correlation analysis between IL-10 expression levels and key biomarkers ACE2, and Arg-1. A Pearson correlation test was used for the association. Red represents positive correlation, blue represents negative correlation, and darker color indicates higher correlation; *P < 0.05, **P < 0.01, ***P < 0.001.

To investigate possible relationships between early pulmonary damage or fibrosis and macrophages polarization status in COVID-19 patients, Pearson correlation analyses were conducted between macrophages M1, M2 type-polarization related cytokines measurements and pulmonary damage or fibrosis biomarkers, as shown in **Figure 4A**. The results were also listed in **Supplementary Tables S1, S2**. Furthermore, the results with correlation coefficients greater than 0.3 in absolute value and statistically significant associations with p < 0.05 were highlighted with scatter plots. Of note, MMP8 exhibited significant positive correlation with Arg-1, VEGF, iNOS, and negative correlation with TNF-α (**Figure 4B**). Additionally, Arg-1 showed positive correlation with ACE2, iNOS, and IL-12 (**Figure 4C**). Furthermore, IL-10 was positively related to ACE2 and Arg-1 (**Figure 4D**). These data demonstrated a significant association between macrophages polarization and pulmonary damage or fibrosis biomarkers.

### 3.6 SP-D and IL-10 as putative biomarkers for predicting severity and pulmonary fibrosis in COVID-19

**Figure 5:**
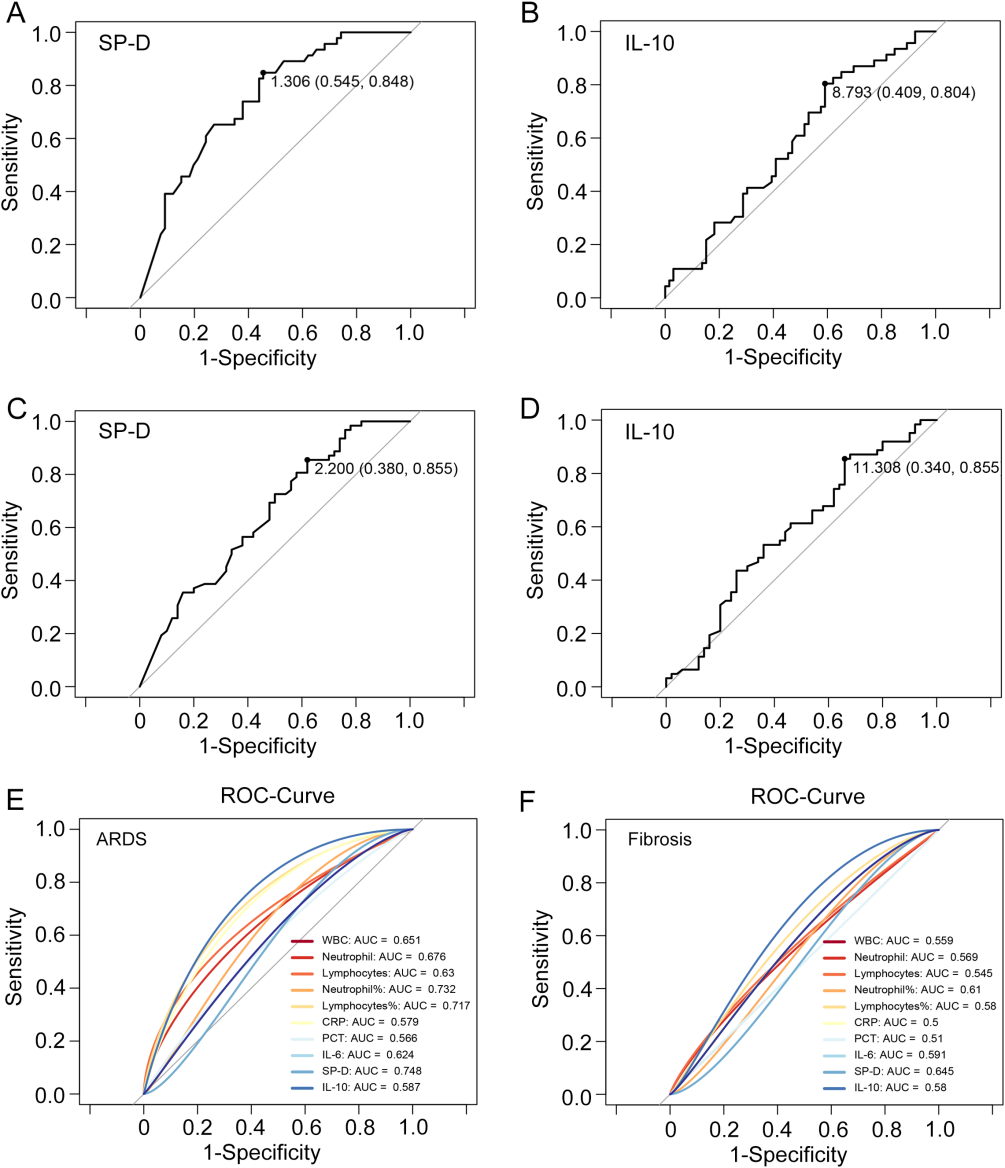
ROC curves of SP-D, IL-10, and main clinical parameters to predict ARDS and pulmonary fibrosis. The ROC curve illustrating the performance of **(A)** SP-D and **(B)** IL-10 to distinguish COVID-19 patients with and without acute respiratory distress syndrome (ARDS). The ROC curve illustrating the performance of **(C)** SP-D and **(D)** IL-10 to distinguish COVID-19 patients with and without fibrosis. **(E, F)** ROC curve analysis of SP-D, IL-10; leukocyte, neutrophil, and lymphocytes subgroup absolute counts; CRP, PCT and IL-6 to predict ARDS and pulmonary fibrosis in patients with COVID-19.

Since SP-D and IL-10 play prominent roles in the progression of COVID-19, the authors believe that their expression levels could reflect disease severity and pulmonary fibrosis progression. The ROC curves were used to evaluate the predictive value of SP-D and IL-10 for severe infection. The results showed that the AUC value of SP-D for predicting severity was 0.748, and the optimal threshold was 1.306, with a sensitivity of 54.54% and a specificity of 84.78% (**Figure 5A**). The AUC value of IL-10 for predicting severity was 0.587, and the optimal threshold was 8.793. It had a sensitivity of 40.90% and a specificity of 80.43% (**Figure 5B**). SP-D achieves higher AUC values than the inflammatory markers concerning severity prediction. Moreover, IL-10 has the similar predictive value as traditional inflammatory indicators, such as CRP and PCT (**Figure 5E**). Next, the significance of SP-D in terms of detecting pulmonary fibrosis progression of COVID-19 was 38.00% sensitivity and 85.48% specificity. The optimal threshold was 2.200, and the AUC was 0.645 (**Figure 5C**). The AUC and optimal threshold were also found for IL-10, with values of 0.580 and 11.308, respectively (**Figure 5D**). The SP-D exhibits significantly higher AUC values compared to other inflammatory markers in terms of predicting pulmonary fibrosis (**Figure 5F**). Collectively, these findings showed that SP-D and IL-10 exhibited potential predictive abilities for the development of severe disease and pulmonary fibrosis, which also indicated that inflammatory and anti-inflammatory imbalance was linked to a poor outcome in SARS-CoV-2 infection.

## 4 Discussion

Patients with COVID-19 are prone to the establishment of irreversible pulmonary fibrosis due to lung tissue damage and impaired gas exchange resulting from SARS-CoV-2-induced interstitial pneumonia and ARDS (19). Fibrotic-like changes, characterized by reticulation, micro-honeycombing, and traction bronchiectasis, were observed in chest CT scans of COVID-19 cases during the acute phase (28). This also suggests a potential association with ARDS as a long-term complication of COVID-19. Moreover, hospitalized patients with milder COVID-19 may also develop post-acute pulmonary fibrosis (29). Therefore, early diagnosis is crucial for proper treatment to avoid aggravation, with biomarkers that reflect lung pathophysiology being more useful in diagnosing the severity of COVID-19 and pulmonary fibrosis.

A dysregulated balance and function of M1 and M2 alveolar macrophages have been linked to the lung pulmonary inflammatory response and fibrosis processes. It is widely acknowledged that during inflammation, the release of inflammatory factors is accompanied by the biosynthesis of specialized anti-inflammatory factors, which could be seen as a negative feedback mechanism to efficiently promote the inflammation shutdown and tissue repair (30). In this study, augmented IL-10 plasma levels were observed in COVID-19 hospitalized patients, with the highest level occurring during the critical phase of the disease (ARDS or pulmonary fibrosis). Importantly, IL-10 was strongly associated with ACE2, which contributes significantly to fibrosis formation and organ remodeling, both of which are features of chronic development after acute inflammation. Thus, IL-10 is a good predictor of disease progression in COVID-19. On the other hand, ARDS is related to the overactivation of macrophages, monocytes, and neutrophils, with the first two also serving as the primary producers of IL-10 (31). Previous studies have reported that IL-10 is an immunoregulatory factor to trigger host immune reaction against viruses, such as influenza virus or respiratory syncytial virus (RSV) (32, 33). Therefore, IL-10, as a promising therapeutic candidate, may also hold promise for the management of ARDS caused by SARS-CoV-2. Of note, the decreased TNF-α in ARDS patients contradicts expected inflammatory patterns. We believe it is related to the use of corticosteroids. The blood samples were obtained subsequent to the short-term corticosteroids therapy for all COVID-19 patients, and all patients were treated with the same type and dose of corticosteroids. The corticosteroids treatment was associated with an early and sustained decrease in TNF-α levels (34, 35).

Alterations in pulmonary surfactant may contribute to the pathogenesis of lung diseases such as ARDS and interstitial lung diseases. The abundant and restricted expression of SP-D within the lungs renders these collections specific markers for various pulmonary disorders. In our study, we observed a significantly higher plasma concentration of SP-D in the ARDS group of COVID-19 patients, as well as in patients who had fibrotic-like changes on chest CT. Considering that pulmonary fibrosis is also a late complication of ARDS, in the acute stage of ARDS, there is a significant increase in SP-D levels attributed to disruption of the air-blood barrier, and excessive proliferation of type II alveolar cells observed in the proliferative stage of ARDS may subsequently result in an overproduction of SP-D (36). In the early stages of SARS-CoV-2 infection, SP-D levels increase due to lung injury-induced increased epithelial barrier permeability and cell loss, thereby representing an indicator for ARDS development. Interestingly, in later stages, the elevation of SP-D appears to protect the lung against viral infections and prevent excessive inflammatory responses. However, in our study, no obvious correlation between SP-D and cytokines associated with macrophage polarization was noticed. It is possible that the elevation of SP-D plasma levels is attributed to the leakage of surfactant proteins into the blood vessels via damaged alveolar capillaries and basement membranes, a process that takes precedence over pulmonary macrophage polarization. This is further supported by the fact that overall expression levels and baseline levels of SP-D were significantly higher compared to most cytokines associated with macrophage polarization. Moreover, the early elevation in circulating levels of SP-D may indicate an adequate response to viral aggression, reflecting the residual capacity and regeneration of surfactant-secreting cells (type II pneumocytes), rather than pulmonary macrophage.

Previous studies have demonstrated that the occurrence and progression of pulmonary fibrosis are facilitated by an increase in local angiotensin Ang-II within lung tissue during acute respiratory distress syndrome, which exerts its effects through the ACE/Ang-II/AT1R pathway (37, 38). Considering that ACE and ACE2 are homologues, the role and mechanism of ACE2 in COVID-19 may be an area for further exploration. ACE2 is a membrane-bound enzyme, and it can be released into the bloodstream through the ADAM17-mediated protein cleavage pathway (39). Our results showed that the expression of ACE2 was significantly up-regulated in COVID-19 patients and exhibits a significant correlation with IL-10. It is suggested that ACE2 could act as one of the main impetuses of macrophage activation and polarization. Emerging evidence demonstrates IL-10 could induce normal alveolar macrophages to express ACE2, making them vectors for SARS-CoV-2, thereby increasing viral susceptibility (40, 41). Meanwhile, in our study, ACE2 was also associated with Arg-1. As a urea cycle-related enzyme catalyzing the conversion of arginine to ornithine or urea, Arg-1 is a hallmark of IL-10-producing immunoregulatory M2 macrophages (42).

Among the COVID-19 patients in this study, 58.9% (66/112) and 44.6% (50/112) have varying degrees of ARDS and fibrotic changes. The level of SP-D was significantly increased in both patients with ARDS and those with fibrosis-like changes on CT scan, suggesting that most patients with ARDS induced by COVID-19 have a higher risk of pulmonary fibrosis compared with patients without ARDS. KL-6 has been shown as a predictive indicator for the risk of secondary pulmonary fibrosis and its reversibility in COVID-19 patients (43). However, in our study, KL-6 did not have a significant predictive value compared to SP-D. Compared to the increase in the lung-specific biomarker KL-6, the increase in SP-A and SP-D initiated at an earlier stage in pneumonia (44, 45). Given that all COVID-19 patients received a short-term corticosteroids therapy before specimen collection, anti-inflammation treatment could alleviate alveolar damage and modulate the overactive immune response. Therefore, the decreased inflammatory exudation will eventually lead to a gradual reduction in KL-6 levels. As pneumonia progressed from mild to severe, there was a significant elevation in the plasma levels of SP-D, which was closely accompanied by clinical observations and CT scans. Additionally, we noted a more robust predictive capacity of SP-D in comparison to classical clinical biomarkers. Therefore, employing plasma SP-D as a biomarker to assess the disease severity or pulmonary fibrosis of COVID-19 presents distinct advantages.

The main limitations of this study are as follows. Firstly, the small sample size evaluated due to the strict classification criteria and the single-center sampling adopted, might increase the standard error of variables. This represents a significant contributing factor to the notable variations observed in patient demographics, including age, gender, and other relevant factors. Secondly, blood samples were collected from patients only at the time of admission. Dynamic monitoring data might expand the applicability of the results obtained here. Thirdly, the time elapsed between the onset of symptoms and sampling was not taken into account due to ambiguity in evaluating initial clinical symptoms of patients with COVID-19. Finally, the lack of adjustment for confounders, such as prior treatment information or primary disease, could lead to residual confounding. The resolution of these problems in the future may provide a better picture of the correlation between these biomarkers and the ARDS severity or pulmonary fibrosis in patients with COVID-19.

## 5 Conclusion

In summary, our study found that SP-D and IL-10 exhibited certain predictive abilities for the development of ARDS and pulmonary fibrosis in patients with COVID-19. The comprehensive evaluation of these biomarkers not only deepens our understanding of disease progression following respiratory viral infections, but it also provide valuable insights into personalized treatment strategies designed to substantially improve the prognosis of patients with post-inflammatory pulmonary fibrosis.

## Data Availability

The original contributions presented in the study are included in the article/**Supplementary Material**. Further inquiries can be directed to the corresponding author/s.
