# Supplementary material for: Increased circulating levels of SP-D and IL-10 are associated with the development of disease severity and pulmonary fibrosis in patients with COVID-19
Source: Front Immunol. 2025 Mar 14;16:1553283. doi: 10.3389/fimmu.2025.1553283 (PMC11949947; doi:10.3389/fimmu.2025.1553283)
Supplement: Supplementary file 1 [file DataSheet1.pdf]

Supplementary Table 1

Relationship between macrophages polarization-related cytokines and pulmonary fibrosis biomarkers during acute COVID-19.

r	SP-D	MMP8	KL-6	ACE2	IL-10	Arg-1	TGF-β	VEGF	iNOS	TNF-α	IL-1β	IL-12
SP-D	1	0.082294	0.005601	0.187313	0.253867	0.130233	-0.09017	0.010631	-0.03036	-0.14769	-0.00161	0.182695
MMP8	0.082294	1	0.130486	0.267776	0.238959	0.439505	0.145971	0.317588	0.540878	-0.40875	0.111774	0.171368
KL-6	0.005601	0.130486	1	0.137666	0.123663	0.103152	0.25353	0.265197	0.010242	0.049939	0.251791	-0.04309
ACE2	0.187313	0.267776	0.137666	1	0.957813	0.350237	-0.07289	0.265984	0.133274	-0.25699	-0.01637	0.06202
IL-10	0.253867	0.238959	0.123663	0.957813	1	0.411047	-0.04321	0.237923	0.154584	-0.23724	-0.02672	0.109964
Arg-1	0.130233	0.439505	0.103152	0.350237	0.411047	1	0.14915	0.255679	0.562776	-0.22867	0.158003	0.446246
TGF-β	-0.09017	0.145971	0.25353	-0.07289	-0.04321	0.14915	1	0.230872	0.064044	-0.06883	-0.11574	0.002019
VEGF	0.010631	0.317588	0.265197	0.265984	0.237923	0.255679	0.230872	1	0.229828	-0.18527	0.039541	0.118876
iNOS	-0.03036	0.540878	0.010242	0.133274	0.154584	0.562776	0.064044	0.229828	1	-0.22193	0.104278	0.290341
TNF-α	-0.14769	-0.40875	0.049939	-0.25699	-0.23724	-0.22867	-0.06883	-0.18527	-0.22193	1	0.078723	-0.07293
IL-1β	-0.00161	0.111774	0.251791	-0.01637	-0.02672	0.158003	-0.11574	0.039541	0.104278	0.078723	1	-0.09296
IL-12	0.182695	0.171368	-0.04309	0.06202	0.109964	0.446246	0.002019	0.118876	0.290341	-0.07293	-0.09296	1

Supplementary Table 2

Relationship between macrophages polarization-related cytokines and pulmonary fibrosis biomarkers during acute COVID-19.

P-value	SP-D	MMP8	KL-6	ACE2	IL-10	Arg-1	TGF-β	VEGF	iNOS	TNF-α	IL-1β	IL-12
SP-D		0.339058	0.948204	0.028395	0.002759	0.129307	0.294694	0.901873	0.724671	0.085023	0.985059	0.032614
MMP8	0.339058		0.128559	0.001559	0.004921	7.74E-08	0.088752	0.000156	8.91E-12	7.09E-07	0.193474	0.045256
KL-6	0.948204	0.128559		0.108665	0.149949	0.230333	0.002796	0.001737	0.90545	0.562235	0.002996	0.617088
ACE2	0.028395	0.001559	0.108665		0	2.72E-05	0.397262	0.001681	0.120525	0.002434	0.849371	0.471542
IL-10	0.002759	0.004921	0.149949	0		6.06E-07	0.61614	0.005116	0.071288	0.005248	0.756639	0.200831
Arg-1	0.129307	7.74E-08	0.230333	2.72E-05	6.06E-07		0.081946	0.002565	8.22E-13	0.007195	0.065178	4.62E-08
TGF-β	0.294694	0.088752	0.002796	0.397262	0.61614	0.081946		0.006642	0.457173	0.424145	0.178034	0.981315
VEGF	0.901873	0.000156	0.001737	0.001681	0.005116	0.002565	0.006642		0.006899	0.0302	0.646408	0.166491
iNOS	0.724671	8.91E-12	0.90545	0.120525	0.071288	8.22E-13	0.457173	0.006899		0.00915	0.225255	0.000578
TNF-α	0.085023	7.09E-07	0.562235	0.002434	0.005248	0.007195	0.424145	0.0302	0.00915		0.360501	0.397027
IL-1β	0.985059	0.193474	0.002996	0.849371	0.756639	0.065178	0.178034	0.646408	0.225255	0.360501		0.279943
IL-12	0.032614	0.045256	0.617088	0.471542	0.200831	4.62E-08	0.981315	0.166491	0.000578	0.397027	0.279943	
